# The effects of paranoia and dopamine on perception of cohesion and conspiracy: a pre-registered, double-blind, placebo-controlled experiment

**DOI:** 10.1007/s00213-023-06476-7

**Published:** 2023-10-17

**Authors:** N.J. Raihani, S.K. Kamboj, M.J. Peniket, J. Norman, O.C. Ozturk, G. Iskandar, V. Bell

**Affiliations:** 1 Department of Experimental Psychology, University College London, 26 Bedford Way, London, WC1H 0AP UK; 2 School of Psychology, University of Auckland, Auckland, New Zealand; 3 Clinical Psychopharmacology Unit, Department of Clinical, Educational & Health Psychology, University College London, 1-19 Torrington Place, WC1E 7HB, London, UK; 4 Department of Clinical, Educational & Health Psychology, University College London, 1-19 Torrington Place, WC1E 7HB, London, UK; 5 Department of Anaesthesia and Perioperative Medicine, UCLH, London, UK; 6 South London & Maudsley NHS Foundation Trust, London, UK

**Keywords:** Paranoia, Conspiracy, Dopamine, Cohesion

## Abstract

Paranoia is a common symptom of psychotic disorders but is also present on a spectrum of severity in the general population. Although paranoia is associated with an increased tendency to perceive cohesion and conspiracy within groups, the mechanistic basis of this variation remains unclear. One potential avenue involves the brain’s dopaminergic system, which is known to be altered in psychosis. In this study, we used large-N online samples to establish the association between trait paranoia and perceptions of cohesion and conspiracy. We further evaluated the role of dopamine on perceptions of cohesion and conspiracy using a double-blind, placebo-controlled laboratory experiment where participants received levodopa or a placebo control. Our results were mixed: group perceptions and perceptions of cohesion were higher among more paranoid individuals but were not altered under dopamine administration. We outline the potential reasons for these discrepancies and the broader implications for understanding paranoia in terms of dopamine dysregulation.

## Introduction

Paranoia is the commonest symptom in psychotic disorders but also exists as a continuous trait in the general population (Bebbington et al. 2013; Elahi et al. 2017; Freeman et al. 2019, 2005). It involves the exaggerated belief that harm will occur and that this is intended by other people (Freeman et al. 2011, 2005). Several studies using participants from the general population have suggested that trait paranoia reflects differences in core socio-cognitive processes, such as the way people attribute intentions to others (Barnby et al. 2022; Greenburgh et al. 2019; Raihani and Bell 2017; Saalfeld et al. 2018) and the value people place upon having positive social interactions (Barnby et al. 2022; Raihani et al. 2021). For example, previous work has shown that paranoia is associated with an exaggerated tendency to attribute harmful intentions to others when their true intentions cannot be decisively determined (Barnby et al. 2020b; Raihani and Bell 2017; Saalfeld et al. 2018) and that paranoia may also be associated with variation in social preferences, such as the self-reported enjoyment of positive or negative social interactions (Raihani et al. 2021). Other work has shown a reduced tendency to cooperate in paranoia (Raihani et al. 2021)—and among patients with schizophrenia (Hanssen et al. 2018)—and has indicated that alterations to reward processing might underpin some variation in social behaviour across the psychosis spectrum (Fett et al. 2019, 2016; Gromann et al. 2014, 2013).

More generally, paranoia has been hypothesised to reflect changes in socio-cognitive processes important for affiliation, perception of coalitions, and the strategic management of relationships (Raihani and Bell 2019). Indeed, paranoia is associated with an increased tendency towards conspiracy thinking (Freeman and Bentall 2017; Greenburgh et al. 2021; Greenburgh and Raihani 2022), defined as the belief that malevolent groups of actors are working together to effect a malign outcome for the self or others (Douglas et al. 2019, 2017). While conspiratorial narratives certainly include attributions of harmful intent, another key component is the perception of coordination among the conspiring individuals. Patients with a clinical diagnosis of psychosis who experience delusions often report being targeted by a group of conspirators, rather than by solo agents (Bell et al. 2020; Raihani and Bell 2019), though these groups are either entirely illusory, or fail to correspond to any group who share the aims and actions attributed to them. This raises the possibility that there is variation along the paranoia spectrum, not just in the perception that others intend harm, but in when and how people perceive group boundaries—and that this perception of group boundaries might underpin at least part of the tendency towards conspiratorial narratives in paranoia.

Despite the recent uptick in work exploring the link between trait paranoia and the tendency to endorse conspiratorial narratives (Freeman et al. 2020; Freeman and Bentall 2017; Greenburgh et al. 2021), far less is known about the mechanistic basis of conspiracy thinking and how this might vary with paranoia. One promising mechanistic avenue involves the brain’s dopaminergic system (Kesby et al. 2018; McCutcheon et al. 2019). Dysregulation of the brain’s dopamine system—involving higher levels of presynaptic dopamine leading to greater binding of dopamine at D2 dopamine receptors—is present in psychosis (McCutcheon et al. 2019). All effective antipsychotic medications are D2 dopamine receptor antagonists (Girgis et al. 2019) and amphetamine, which acts to increase D2 dopamine receptor binding, causes psychosis in amphetamine users in the community (Arunogiri et al. 2020). Conversely, antipsychotic medication operates by ameliorating the effects of existing dopaminergic dysfunction or tempering excessive release of dopamine (Sommer et al. 2012). Although the development of psychosis is known to depend upon several disparate social, genetic and environmental stressors, it has been suggested that these various insults, accumulated during the lifespan, converge in a ‘final common pathway’, mediated by the subcortical dopamine system (Howes and Kapur 2009), which results in the characteristic positive symptoms associated with psychosis.

Initial studies have shown manipulations of the dopamine system can affect social interactions, including affecting trust judgements (Bellucci et al. 2020), moral decision-making (Crockett et al. 2015) and generosity (Artigas et al. 2019). Comparative work suggests that dopamine’s broad role in social interactions is likely to be evolutionarily conserved, being evident in a disparate range of vertebrate taxa (Antunes et al. 2022; Skuse and Gallagher 2009). A core feature of human social cognition is deciding who falls into the category of ‘us’ versus ‘them’ and it is possible that dopamine impinges upon such recognition and classification processes (Shkurko 2015). This suggests that dopamine may regulate how we perceive social groups and that this may in part explain variation in perception of and responses to social groups in paranoia. We test this hypothesis here.

We test two main hypotheses: (1) paranoia involves alterations to the perception of group boundaries and an increased perception of conspiracy and (2) altering dopamine functioning of healthy participants through safe, lab-based drug administration of levodopa (the precursor for the catecholamine neurotransmitters dopamine, norepinephrine and epinephrine) will temporarily alter the perception of group boundaries in a similar way to paranoia. To test these ideas, we used (i) large-N online behavioural experiments and (2) lab-based studies where we conducted dopaminergic manipulations using levodopa.

## Methods

This study was approved by the UCL Research Ethics Committee under the project 3720-002. Participation was voluntary and participants gave informed consent prior to taking part. For both online and lab-based studies, we recruited participants who reported having no recent history of mental ill health from the general population. Paranoia shows full taxometric continuity across clinical and non-clinical samples (Elahi et al. 2017), indicating general population samples can yield insights into more severe paranoia typically observed in clinical settings.

Participants in both the online and the laboratory studies performed two tasks measuring perception of group cohesion and perception of conspiracy (described below). Participants also took part in additional experiments not reported here which form the basis of other studies investigating the effects of paranoia and L-DOPA administration on other aspects of social cognition. The predictions for both the online and the laboratory studies were pre-registered (pre-registrations, data and code associated with this study can be found at https://osf.io/vgy6e/). We report any deviations from the pre-registered hypotheses or analyses below.

### Recruitment

#### (i) Online

Data were collected in November 2021. We initially recruited 1553 UK-based participants via the online recruitment platform Prolific.ac, though data from 50 participants were subsequently excluded for speeding through tasks too quickly or for failing an attention check (as per the pre-registration). This left us with a sample of 1503 for analysis. The age range of this sample was 16–88 years old (mean = 40.2 ± 0.4). Of the 1503 participants, 941 were female, 547 were male and the remainder either identified as non-binary or preferred not to disclose their gender. Participants initially completed the Revised Green et al. Paranoid Thoughts Scale (R-GPTS, Freeman et al. 2019) and some other tasks measuring perception of cohesion in groups (see below). They were subsequently recalled after a minimum interval of 4 days to take part in the Cyberball task (described below) and another task not presented here. At this stage, we successfully recalled 679 of the original 1503 eligible participants and this is the sample size available for the Cyberball (online sample) study.

#### (ii) Lab

Data were collected from April to December 2022. Participants were recruited via a departmental database and through online advertising. Because the lab study involved dopamine administration, our inclusion criteria were more stringent than for the online study (for safety reasons). Specifically, to be eligible to participate in the study, participants had to meet certain criteria. These included being between 18 and 45 years old, not having any neurological issues (such as seizures), not currently requiring treatment for a mental health problem, not having any type of skin cancer, not having any heart problems or abnormal heart rhythms, having a resting heart rate of over 60 bpm, not having any stomach or gastrointestinal ulcers, not experiencing dyskinesia, being able to fully empty their bladder, not having any liver problems, not having glaucoma and not having lactose or dairy intolerance. Participants were also excluded if they were daily smokers, took potent inhibitors of CYP3A4, if they took drugs that prolong the QT interval, if they were breastfeeding or pregnant and if they regularly took recreational drugs other than alcohol, caffeine and nicotine or consumed more than 14 units of alcohol per week. The online and phone screening surveys that participants completed are available at https://osf.io/vgy6e/.

Initially, 187 UK-based participants opted into the online screening phase of the study. Of these, 66 individuals passed both the online and phone screening checks and attended at least one experimental session in the lab. Subsequently, data from 6 participants were excluded because participants experienced nausea and vomiting after the levodopa (hereafter, L-DOPA) administration. This left us with a sample of 60 participants who attended both experimental sessions and experienced no nausea (40 females, 20 males). This met our pre-registered target sample size. The age range of this sample was 18–45 years old (mean = 23.5 ± 0.7).

### Drug administration (lab)

We wanted to explore the effect of elevated dopamine on various aspects of social cognition. To that end, participants took part in two experimental sessions where they either received L-DOPA with an antiemetic (see below for details) or a vitamin C placebo with the same antiemetic. L-DOPA was administered as co-beneldopa capsules (Madopar: Roche Products Ltd.) which contained 12.5/25 g bensearazide (a DOPA decarboxylase inhibitor) and 50/100 mg L-DOPA. Drug and placebo capsules were matched in size and number, and administration was double-blind and fully randomised across participants.

We originally planned to administer 300 mg L-DOPA (alongside 10 mg domperidone). However, early in the study it became clear that this dose was poorly tolerated (due to nausea and vomiting). Two steps were taken to obviate adverse effects. Firstly, the L-DOPA dose was adjusted downwards, first to 200 mg and then 150 mg. As such, of the 60 participants, 42 received 150 mg L-DOPA; 17 received 200 mg and 1 participant received 300 mg L-DOPA. Secondly, the co-administered antiemetic was changed from 10 mg domperidone (*n* = 16) to the more potent ondansetron (8 mg; *n* = 43). Due to the unanticipated variation in L-DOPA dose received by participants, we checked that all analyses reported below were robust to the exclusion of the data from the 18 participants who received more than 150 mg L-DOPA. All results below are robust to the exclusion of these data points and are based on the full sample of 60 participants.

## Procedure

To establish the physiological effects of L-DOPA administration, lab participants first watched a 10-min YouTube video (showing ‘World’s Most Beautiful Railway’) where we measured their spontaneous eye blink rate (sEBR), which is typically enhanced under elevated dopamine (Jongkees and Colzato 2016; Van Slooten et al. 2019 but see Sescousse et al. 2018). As four videos were slightly shorter than 10 min (due to internet connection problems or adverts interrupting the video), we calculated the number of spontaneous eye blinks per second of video watched under both treatment and placebo conditions. Data from four participants were excluded from the analysis as no video data were available for one of the experimental sessions. Although sEBR rates were higher under L-DOPA (0.32 blinks/s compared with 0.29/s under placebo), this difference was not significant at conventional levels (paired *t*-test, *t* = −1.61, DF = 54, *p* = 0.11).

For both online and lab participants, the tasks and assessment measures were similar. All participants completed the R-GPTS, which measures ideas of social reference and persecution over the previous month. Participants rate their agreement with 18 statements (8 measuring social reference, 10 measuring persecution) on a scale of 0 to 4, with higher scores indicating stronger agreement. As per our pre-registration, we used the persecution subscale in our analyses as a proxy for paranoid ideation.

**Fig. 1:**
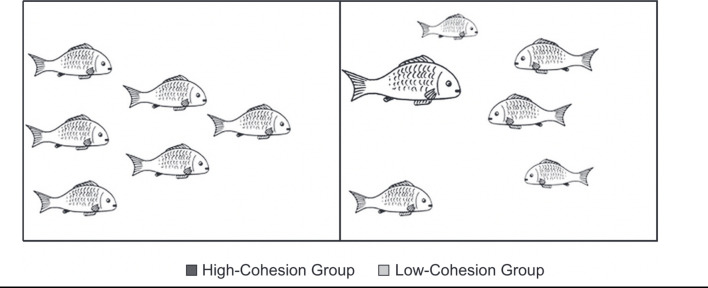
The high-cohesion and low-cohesion fish shoals observed by participants for the cohesion perception task, reproduced from Waytz and Young (2012)

Participants subsequently completed two key tasks to measure social cognition: a *cohesion perception* task and a *Cyberball* task. The *cohesion perception* task was derived from Waytz and Young (2012) and measured perceptions of cohesion and the extent to which group members are perceived to have an individual versus a group mind. Participants watched two 44-s videos of animated fishes of the same size that either swam in a highly coordinated or an uncoordinated manner (Fig. 1, order counterbalanced). As per Waytz and Young (2012), following each video, participants were asked to rate on a 7-point Likert scale (where 1 = not at all and 7 = very much) (i) the extent to which they thought each individual fish had a mind; (ii) how cohesive the group of fish were; and (iii) the extent to which the group of fishes had a mind.

*Cyberball* is a computerised ball-throwing game that can be used to measure perception of and responses to social exclusion (Hartgerink et al. 2015; Williams and Jarvis 2006). Participants played two *Cyberball* games (experiment was created and hosted on Gorilla Experiment Builder, www.gorilla.sc) with three other (fictitious) players. Co-player identities were changed across games. Each game comprised 30 ball throws in total. In the ‘inclusion’ game, the participant received the ball with 0.25 probability whereas in the exclusion game the participant received the ball exactly once from every other player and did not receive the ball again thereafter. Following each game of *Cyberball*, participants were asked to rate the extent to which they agreed or disagreed with three statements using a 5-point Likert scale, where higher numbers indicated stronger disagreement. The statements were as follows: (i) The other players knew one another; (ii) the game was unpleasant; (iii) the other players deliberately excluded me from the game. For ease of interpretation, we subsequently recoded these responses so that higher scores indicate higher agreement*.* The order in which participants played the inclusion and exclusion games was counterbalanced across subjects and participants were fully debriefed that the other players were not real at the end of each experimental day.

## Pre-registered predictions

We describe the key pre-registered predictions (and any deviations from these) below. All analyses were performed in R Studio (version 4.2.2).

*Cohesion perception task:* We first aimed to replicate the basic findings from Waytz and Young (2012), namely that (1) perceptions of group cohesion and group mind would be higher for the high-cohesion than for the low-cohesion fish and (2) perception of individual group member minds would be higher for low-cohesion than for high-cohesion fish. Replicating Waytz and Young (2012), we also expected to observe (3) positive correlations between perception of cohesion and group member mind and (4) negative correlations between perception of group mind and individual member mind.

To test these predictions, we used the full sample from the online data set and the data from participants under the placebo condition from the lab data set. We report one deviation from our pre-registered analyses here because we failed to specify whether we would use data from the low-cohesion or the high-cohesion fish videos to run the correlation analyses (3 and 4). We present data for the high-cohesion fish below and report any qualitative differences when running the same tests on the low-cohesion fish in the text.

Going beyond previous work, we further expected perception of cohesion and perception of group mind to be modified by trait paranoia (online sample) and by L-DOPA administration (lab sample), such that more paranoid or dopamine-exposed participants would perceive even greater cohesion and more of a group mind among the high-cohesion fishes. In the online sample, this prediction was tested by setting cohesion/group mind as an ordinal categorical response term in a cumulative link model using package brms (Bürkner 2017), with the following explanatory variables: condition (high-cohesion/low-cohesion), paranoia and the two-way interaction between these. Paranoia score was standardized by subtracting the mean and dividing by twice the standard deviation (Gelman 2008). We also included participant ID as a random term to account for the fact that we had two data points per participant in the model.

In the lab sample, we pre-registered a Wilcoxon-signed rank test of perception of cohesion under L-DOPA compared to placebo using data from the high-cohesion fish only. To better match the analyses for the online data analyses above, we also performed similar unregistered analyses on the low-cohesion fish, and we also investigated whether perceptions of group mind varied under L-DOPA compared to placebo in both the high-cohesion and the low-cohesion conditions.

*Cyberball task:* To establish that participants experienced the inclusion and exclusion conditions differently, we predicted that perception of exclusion, experiencing the game as unpleasant, and believing that the other players knew one another would be higher in the exclusion compared to the inclusion condition, in both the online and lab participants.

For the lab participants, we tested whether perceptions of exclusion, experiencing the game as unpleasant, and believing that the other players knew one another were higher in the exclusion compared to the inclusion condition. We tested these predictions separately in the L-DOPA and the placebo data, using paired data from day 1 for each participant. We neglected to include a prediction that believing the other players knew one another would be higher under exclusion than inclusion in our pre-registration for the lab sample but we include the two tests (one for L-DOPA, one for placebo) in the results section for completeness.

We were mainly interested in whether perceptions of exclusion and conspiracy would be associated with trait paranoia (online sample) or L-DOPA administration. To test this in the online data, we used two ordinal regression models (run with package brms as above) to determine whether perceptions of exclusion and conspiracy, respectively, were higher among more paranoid participants, and whether there was a paranoia x condition interaction, such that more paranoid individuals would report higher feelings of exclusion and conspiracy in the exclusion Cyberball game.

For these tests, we note an important deviation from our pre-registered analyses concerning the lab data. In the lab study, we informed participants at the end of day 1 that their co-players in the game were fictitious. This debriefing (which was performed for ethical reasons) raised concerns, both for us and the reviewers of this paper, about the validity of the day 2 data, since participants might have remembered that the co-players in the *Cyberball* game were not real. To address this, we decided to restrict all *Cyberball* analyses for the lab participants to day 1. As some participants received L-DOPA on day 1, while others received placebo, this decision meant that we could no longer perform our pre-registered paired analysis of how perceptions of exclusion and conspiracy varied under L-DOPA compared to placebo. Instead, we used two-sample Wilcoxon tests to ask whether perceptions of exclusion and conspiracy, respectively, were higher for participants who received L-DOPA (compared to placebo). Our pre-registration indicated that we would compare data in the exclusion condition, but, to better match the predictions to those tested with the online sample, we also tested whether perceptions of conspiracy and exclusion were higher under L-DOPA in the inclusion condition.

## Results

### R-GPTS scores

The scores for social reference and persecution for the online and lab samples are shown in Table 1, along with the number of participants falling into each descriptive category (according to categorical thresholds defined in Freeman et al. 2019). Most participants fell within the ‘average’ category for social reference and persecution, as expected. In the lab sample, social reference scores ranged from 0 to 19 and persecution scores from 0 to 23. In the online study, social reference scores ranged from 0 to 32 and persecution scores from 0 to 40. Therefore, the online study covered the full spectrum for paranoid ideation while there was a more limited range of paranoia scores among our laboratory participants.

**Table 1:** Number of participants from the lab and online samples who fell within each of the five categories of severity for social reference and persecution thoughts (classifications based on Freeman et al. 2019)

	Sample	Average	Elevated	Moderately severe	Severe	Very severe	Mean score
Social reference	Lab	45	12	3	0	0	5.72 ± 0.65
	Online	1044	250	125	57	27	7.27 ± 0.17
Persecution	Lab	48	8	1	3	0	2.8 ± 0.62
	Online	1008	228	122	108	37	5.22 ± 0.20

Standard errors are reported with mean data

### Cohesion perception task

People perceived the high-cohesion fish as being more cohesive (*Online*: Wilcoxon signed rank test, *V* = 949,778, *p* < 0.001; *Lab*: *V* = 1768, *p* < 0.001) and having more of a group mind than the low-cohesion fish (*Online*: Wilcoxon signed rank test, *V* = 457,175, *p* < 0.001; *Lab*: *V* = 983, *p* < 0.001; Table 2). Similarly, perceptions of individual fish having their own mind was stronger for the low-cohesion than the high-cohesion fish (*Online:* Wilcoxon signed rank test, *V* = 21,444 *p* < 0.001; *Lab: V* = 5, *p* < 0.001).

**Table 2:** Mean (± sem) ratings for statements asking whether each fish had an individual mind, whether the shoal formed a cohesive group and whether the shoal had a group mind

	Online sample (*n* = 1503)	L-DOPA (*n* = 60)	Placebo (*n* = 60)
To what extent does each individual fish have a mind?			
Coordinated fish	2.89 ± 0.05	2.63 ± 0.18	2.8 ± 0.20
Uncoordinated fish	4.88 ± 0.04	5.28 ± 0.18	5.43 ± 0.17
How cohesive was the group of fishes?			
Coordinated fish	6.41 ± 0.03	6.23 ± 0.16	6.3 ± 0.12
Uncoordinated fish	3.42 ± 0.04	2.28 ± 0.15	2.33 ± 0.13
To what extent does the group of fishes have a mind?			
Coordinated fish	4.45 ± 0.05	4.4 ± 0.21	4.67 ± 0.21
Uncoordinated fish	3.77 ± 0.04	3.02 ± 0.19	3.07 ± 0.20

Ratings were measured using a 7-point Likert scale, where 1 = ‘Not at all’ and 7 = ‘Very much’

As expected, we also found positive correlations between perceptions of cohesion and group mind in both samples (Spearman’s rank correlation *Online*: *p* < 0.002, rho = 0.26; *Lab*: *p* = 0.003, rho = 0.38). In the online sample, perception of shoal cohesion was negatively correlated with individual mind perception, as expected (*Online:* Spearman’s rank correlation, *p* = 0.02, rho = −0.06). Nevertheless, we did not find a corresponding significant correlation in the lab sample (*p* = 0.92, rho = −0.01). Counter to predictions (and counter to Waytz and Young 2012), there was a significant, positive correlation between perceptions of individual and group mind in the online sample (Spearman’s rank correlation, *p* < 0.001, rho = 0.30) whereas a non-significant negative correlation was observed in the lab sample (*p* = 0.38, rho = −0.12).

In the online sample, trait paranoia was negatively related to perceptions of cohesion (estimate: −0.11, credible intervals: −0.23, 0.01), which was counter to our expectations, though the credible intervals included zero which is also consistent with a null effect. As expected, high-cohesion fish were perceived as being more cohesive (estimate: −2.21, CI: −2.11, −2.32) but the interaction between paranoia and condition revealed no effect on perceptions of cohesion (estimate: 0.13, CI: −0.03, 0.29).

**Fig. 2:**
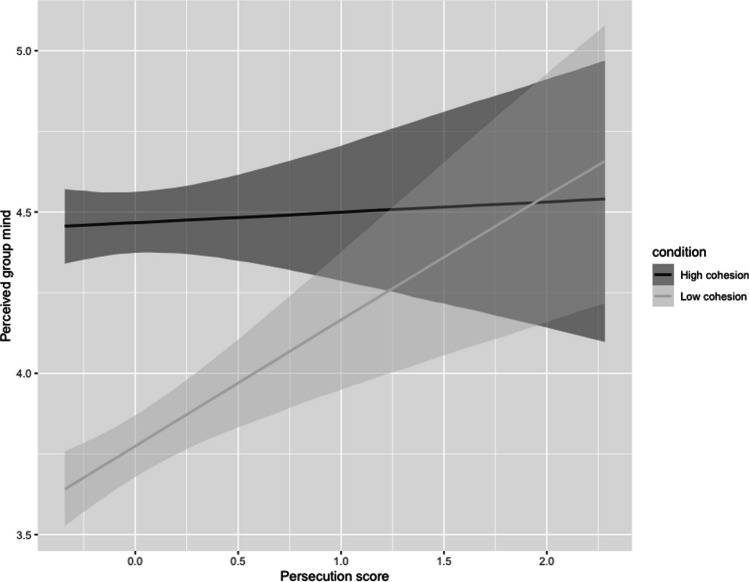
Conditional effects plot showing how perception of group mind varies with trait paranoia score (persecution subscale) and cohesion condition (high/low). Plot shows posterior predicted distribution with 95% credible intervals of the mean

The results for perception of group mind were a little different. While the high-cohesion fish were perceived as having more of a group mind, as expected (estimate: −0.43, CI: −0.35, −0.50), we also found a significant interaction between trait paranoia and fish cohesion on perception of group mind (estimate: 0.22, CI: 0.07, 0.37). Specifically, more paranoid individuals were more likely to perceive the low-cohesion shoal as having a group mind (Fig. 2).

We found no significant difference in perception of group cohesion under L-DOPA compared to placebo, either for the high-cohesion fish (Wilcoxon signed-rank test, *V* = 170, *p* = 0.89) or for the low-cohesion fish (*V* = 226.5, *p* = 0.47). Similarly, we detected no significant difference in perception of group mind under L-DOPA compared to placebo, either for the high-cohesion fish (*V* = 317, *p* = 0.13) or the low-cohesion fish (*V* = 532.5, *p* = 0.93).

### Cyberball task

Participants felt more excluded in the *Cyberball* exclusion condition than the inclusion condition (Wilcoxon test, Online: *V* = 802, *p* < 0.001; Lab *placebo*: *V* = 0, *p* < 0.001, Table 3), found it more unpleasant (Online: *V* = 2283, *p* < 0.001; Lab *placebo*: *V* = 14, *p* < 0.001, Table 3) and were more likely to think the other players knew one another (Online: *V* = 3688.5, *p* < 0.001; Lab *placebo*: *V* = 12, *p* < 0.001, Table 3). All pairwise tests reported here were also significantly different in the L-DOPA condition for the lab sample.

**Table 3:** Mean (±sem) perceptions of exclusion, feelings of unpleasantness and perceptions of conspiracy among the co-players in the *Cyberball* task

	Online sample (*n* = 1503)	L-DOPA (*n* = 30)	Placebo (*n* = 30)
Felt excluded			
Inclusion	1.77 ± 0.03	1.93 ± 0.14	1.93 ± 0.16
Exclusion	3.73 ± 0.04	3.97 ± 0.15	4.07 ± 0.14
Found the game unpleasant			
Inclusion	1.98 ± 0.03	2.37 ± 0.16	2.47 ± 0.16
Exclusion	3.03 ± 0.04	3.53 ± 0.15	3.20 ± 0.19
Thought other players knew one another			
Inclusion	2.42 ± 0.03	3.10 ± 0.16	2.87 ± 0.16
Exclusion	3.16 ± 0.04	3.73 ± 0.17	3.73 ± 0.17

We only report data from day 1 of the study before participants were debriefed about the co-players in the game being fictitious. Scores represent agreement with the statements about the game and were  measured using a 5-point Likert scale, with higher scores indicating stronger agreement with the statements. Data are shown for the inclusion and exclusion conditions of the game. All pairwise differences between the inclusion and exclusion conditions in this table are significant at the 0.05 level

In the online sample, perceptions of exclusion were higher among more paranoid individuals (estimate: 0.29, CI: 0.09, 0.49) though there was no interaction between trait paranoia and condition (estimate: −0.19, CI: −0.43, 0.05), such that more paranoid individuals did not feel especially excluded in the exclusion or the inclusion game. Perceptions of conspiracy were positively associated with paranoia, though credible intervals included zero, indicating that a null effect was also possible (estimate: 0.12, CI: −0.12, 0.37). Counter to our predictions, there was no evidence for a paranoia x condition interaction on perceptions of conspiracy (estimate: −0.06, CI: −0.29, 0.18).

Perceptions of exclusion were not affected by L-DOPA, either in the inclusion condition (Wilcoxon rank-sum test, *W* = 438, *p* = 0.85) or the exclusion condition (*W* = 428, *p* = 0.72). Similarly, perceptions of conspiracy among the co-players were unaffected by L-DOPA, either in the inclusion (*W* = 395.5, *p* = 0.39) or the exclusion (*W* = 450, *p* = 1) condition.

## Discussion

We aimed to test whether trait paranoia was associated with perceptions of group boundaries, using both an abstract visual experiment as well as a more socially salient experimental paradigm. We further aimed to tie any paranoia-mediated variation in group perception to a well-evidenced mechanistic pathway by examining whether dopaminergic manipulations would produce similar results as those observed among the high-paranoia online sample. Our results were mixed. Trait paranoia was associated with group perception (and specifically the perception of a group mind) and also seemed to predict stronger perceptions of exclusion in the *Cyberball* game. Nevertheless, paranoia was not robustly associated with perceptions of conspiracy, and the L-DOPA manipulations did not accord with the paranoia-based associations we uncovered, in that perceptions of group mind were not elevated under L-DOPA compared to placebo, and feelings of exclusion in the *Cyberball* task (which were elevated in those with high paranoia) were unlinked to L-DOPA administration in the lab sample.

Together, there was partial support for our pre-registered hypothesis that paranoia is associated with variation in group perception but no support for the hypothesis that any such variation can be understood in terms of dopaminergic dysregulation in the brain. Our study suffered from some important limitations which may have hindered our ability to detect any dopamine-linked effects on cognition. One limitation is that we were forced to administer a much lower dopamine dose than planned due to low tolerability of the higher doses in our participants. While previous work has also reported using a 150 mg dose of L-DOPA (e.g. Barnby et al. 2020a; Crockett et al. 2015), it is possible that this dose (which was received by most participants in this study) was insufficient to generate the size of effects we could reliably detect with a relatively small sample size of 60 participants. Indeed, we did not observe a significantly elevated sEBR under L-DOPA (though there is some uncertainty pertaining to the robustness of the relationship between striatal dopamine and sEBR, (Sescousse et al. 2018)). While we cannot be sure about the impact of the 150 mg L-DOPA dose, the presence of adverse effects in some participants indicates that, at least for the 200 mg dose, L-DOPA was likely to be exerting some physiological effect.

Another limitation of our study design stems from the fact that lab participants took part in all experiments twice, which may have affected some responses on the second test day. This concern was especially acute for the *Cyberball* study where, due to the requirements of our ethical approval, we debriefed participants that the co-players in the game were not real at the end of each experimental day. Following reviewer comments, we subsequently limited our Cyberball analyses to day 1 data only, meaning that we did not have paired data for the key tests of interest (whether perceptions of exclusion and conspiracy were higher under L-DOPA compared to placebo).

It is also possible that our alteration of the dopaminergic system via L-DOPA administration failed to exert sufficiently large sample-wide effects on the relevant dopaminergic function in the brain. There are several possible reasons for this. Psychosis has largely been associated with dopaminergic dysregulation mediated by D2 dopamine receptors in the striatum, and dopaminergic agents which are either antipsychotic or raise the risk of psychosis (e.g. amphetamine) primarily work by acting as antagonists or agonists on these receptors, respectively. L-DOPA is a non-specific agonist and may have its primary action through D1 receptors (Viaro et al. 2021), meaning it may not have sufficiently altered the D2-mediated striatal dopamine pathway hypothesised to be a key causal mechanism. We note that in a similar previous study involving manipulation of the dopaminergic system to explore effects on social cognition (Barnby et al. 2020a), the predicted alterations in attributions of harmful intent were only observed among participants who received the dopamine antagonist haloperidol and not those who received L-DOPA. Haloperidol is more selective for D2 receptors than L-DOPA, potentially suggesting that this selectivity is needed for paranoia-relevant effects.

Another possibility concerns the validity of the paradigms we used to measure perceptions of group cohesion, exclusion and conspiracy. In particular, we note the abstract nature of the fish task, which may not have been socially salient enough for us to engage paranoia-relevant cognition. Moreover, we did not reliably replicate a key finding from Waytz and Young (2012), that perceptions of group mind should be negatively correlated with perceptions of individual member mind, which calls into question the robustness of this particular paradigm. *Cyberball* is a well-evidenced paradigm that reliably induces feelings of exclusion (Hartgerink et al. 2015; Mwilambwe-Tshilobo and Spreng 2021) and has also been used in people diagnosed with, or at high risk of developing, a psychotic-spectrum disorder (Gradin et al. 2012; Lincoln et al. 2021, 2018; Pillny and Lincoln 2020). Nevertheless, previous evidence is quite mixed, with the overall picture suggesting that feelings of exclusion in response to the *Cyberball* task are not associated with psychosis-proneness (Lincoln et al. 2021). For example, some work suggests blunted responses to social exclusion in schizophrenia (Gradin et al. 2012), whereas other work finds reduced positive emotion in the *inclusion* condition in schizophrenia (Engel et al. 2016). More recent work has shown that psychosis-proneness does not predict perceptions of exclusion, but that exclusion does lead to an increase in state paranoia immediately following the *Cyberball* game (Lincoln et al. 2018; Sundag et al. 2018). In our study, we did find a positive association between trait paranoia and perceptions of exclusion, though this was not especially pronounced in the exclusion condition (which seems to fit with the overall picture described above, as well as the conclusions of a recent systematic review, (Lincoln et al. 2021)).

However, we also consider the possibility that this study provides evidence for the lack of dopaminergic involvement in perceptions of groups, exclusion or conspiracy. Paranoia was related to feelings of exclusion and group mind perception in our tasks, suggesting that these processes are associated with distorted social perceptions in paranoia. However, there were no differences in the L-DOPA condition, which raises the possibility that dopaminergic functions (and specifically dopamine-related learning and reward-seeking mechanisms, Guitart-Masip et al. 2014; Kroemer et al. 2019; Pessiglione et al. 2006, although see Grimm et al. 2021), are not mechanistic components of these processes. It is worth noting the danger of treating neurotransmitters as if they are independent mechanistic components of an overall system, rather than a dynamic complex system that interacts and regulates within and across neural pathways (Sarter et al. 2007). However, previous authors have speculated whether mechanisms more heavily dependent on serotonergic mechanisms may be more central to group processes (Schafer and Schiller 2022).

We note here an alternative hypothesis that has been previously raised in the psychosis literature, namely, that reduced, rather than increased, dopamine synthesis may underlie impairments in social cognition that may account for the negative symptoms of schizophrenia (e.g. (Lewandowski et al. 2016)). However, we also note that the evidence here is complex: striatal dopamine has been associated both negatively (Wong et al. 2022) and positively (McCutcheon et al. 2020) with negative symptoms, and most studies have not measured social cognition directly. Given this, we consider this an open question that requires further investigation.

We note some further limitations with the study design that might have impinged on the results. One exclusion criterion for our study was participants who regularly took recreational drugs or consumed more than a certain threshold of alcohol per week. Nevertheless, this exclusion criterion was based on self-report and our sample is therefore likely to have included some people who ought to have been excluded. For example, in Barnby et al. (2020a), around 20% of people who reported not taking recreational drugs were subsequently excluded on the basis of drugs being detected in a urine sample. We further note that the exclusion criteria for the lab sample were necessarily more restrictive than for the online sample—and that our lab sample was on average younger than the sample we recruited online. It is therefore possible that some characteristics of the lab and online samples, respectively, may have contributed to different findings across these samples.

In summary, we report some selective effects of trait paranoia on perceptions of group cohesion and feelings of exclusion in a social ostracism task. These effects were not replicated in participants who received L-DOPA, suggesting that the associations between paranoia and group perception and feelings of exclusion are not mediated by dopaminergic dysfunction. To be certain in this conclusion, however, would involve testing with an antagonist (e.g. haloperidol) and also with larger L-DOPA doses than we were able to in this study.
